# Phosphogluconate dehydrogenase is a predictive biomarker for immunotherapy in hepatocellular carcinoma

**DOI:** 10.3389/fonc.2022.993503

**Published:** 2022-10-20

**Authors:** Tiantian Liu, Jianni Qi, Hao Wu, Le Wang, Lihui Zhu, Chengyong Qin, Jiao Zhang, Qiang Zhu

**Affiliations:** ^1^ Department of Gastroenterology, Shandong Provincial Hospital, Shandong University, Jinan, China; ^2^ Shandong Provincial Engineering and Technological Research Center for Liver Diseases Prevention and Control, Jinan, China; ^3^ Central Laboratory, Shandong Provincial Hospital, Shandong University, Jinan, China; ^4^ Central Laboratory, Shandong Provincial Hospital Affiliated to Shandong First Medical University, Jinan, China; ^5^ Department of Infectious Disease, Shandong Provincial Hospital Affiliated to Shandong First Medical University, Jinan, China; ^6^ Department of Health Gastroenterology, Shandong Provincial Hospital Affiliated to Shandong First Medical University, Jinan, China; ^7^ Department of Gastroenterology, Shandong Provincial Hospital Affiliated to Shandong First Medical University, Jinan, China

**Keywords:** hepatocellular carcinoma, immunotherapy, immune cell, methylation, phosphogluconate dehydrogenase

## Abstract

**Background:**

Phosphogluconate dehydrogenase (PGD) is involved in the regulation of various tumors. However, its role in hepatocellular carcinoma (HCC) is poorly understood. This study tried to determine the prognostic efficacy of PGD and its value for immunotherapy in HCC.

**Methods:**

The data from the TCGA database was used to explore the predictive power of PGD expression and methylation on the overall survival (OS) of HCC through Cox regression and the Kaplan-Meier analysis. Then, we used the GEO and ICGC database to further verify the predictive power. Finally, the relationship between PGD and immune cells and the relationship between PGD and the efficacy of immunotherapy were explored through bioinformatics analysis in HCC.

**Results:**

PGD is highly expressed in HCC tissues, which is negatively regulated by PGD methylation. Low PGD expression and PGD hypermethylation predict better OS in HCC patients. Besides, a meta-analysis based on the TCGA, GSE14520, and ICGC databases further confirms that low PGD expression is closely related to favorable OS. Then, we find significant differences of immune cell infiltrations between high and low PGD expression groups. Expressions of immune checkpoints, most HLA members and tumor mutation burden (TMB) are higher in the high PGD expression group, which indicates beneficial efficacy of immunotherapy in this group. And the potential mechanisms of PGD are exhibited.

**Conclusion:**

PGD is an independent prognostic factor of HCC patients and plays an important role in immune cell infiltration and immunotherapy, which indicates that PGD can be used as a predictive biomarker for HCC immunotherapy.

## Introduction

Hepatocellular carcinoma (HCC) is one of the most common cancers globally and the third leading cause of cancer-related deaths (1). Epidemiological reports indicate that the incidence of HCC shows gender differences. The incidence of HCC in men is higher than that in women, and the possibility of HCC in men is 2-4 times higher than that in women (2). Nowadays, the most widely used treatment for HCC is surgical resection and liver transplantation. However, these two methods show obvious disadvantages, including postoperative recurrence and liver donor shortage. In addition, these two methods show limitations in the treatment of advanced HCC, so the therapeutic effect of HCC is far from satisfactory (3). Due to the limited curative effect of the treatment for hepatocellular carcinoma, researchers have focused on new methods for treating HCC, among which immunotherapy is a research focus (4, 5).

Phosphogluconate dehydrogenase (PGD), also called 6-phosphogluconate dehydrogenase (6-PGD), plays a key role in the pentose phosphate pathway (PPP) as an oxidative carboxylase (6, 7). In recent years, the overexpression of PGD in various cancers has attracted researchers’ attention (8). Previous studies have reported the up-regulation of PGD in kinds of human cancers, such as cervical cancer (9), ovarian cancer (10), lung cancer (11), and so on. Chen H et al. suggested that PGD was up-regulated in HCC tissue (12). However, there is no research on the clinical and prognostic analysis of PGD in HCC.

Immunotherapy brings new opportunities and developments for tumor therapy (13). However, it is currently found that many factors may affect the effect of immunotherapy, so it is particularly important to find new biomarkers that can predict the effect of immunotherapy (14, 15). Daneshmandi S et al. uncovered that PGD could act as a regulator in the process of CD8+ T cell activation and differentiation, suggesting that PGD might serve as a key therapeutic target for cancer immunotherapy (16). And the same team also found that blocking PGD in the process of oxidative PPP could lead to a significant decrease in the inhibitory function of Tregs and a transition to Th1, Th2, and Th17 phenotypes, proving that PGD played a key role in regulating Treg, thus revealing that PGD could become a new metabolic checkpoint for immunotherapy (17).

In view of the lack of clinical studies on PGD-related HCC, it is meaningful to explore the immune relevance and prognostic value of PGD, as well as the relationship between PGD and the prediction of immunotherapy effects.

In this study, the differences of PGD expression in HCC tissues and adjacent tissues were compared, and the significance of PGD on the prognosis of HCC was studied. Subsequently, we further integrated the HCC cases in the three databases (TCGA, ICGA, GSE14520) through meta-analysis to verify the prognostic ability of PGD. We also evaluated the relationship between PGD and tumor immune cell infiltration and then explored the relationship between PGD and the efficacy of immunotherapy. Finally, we conducted functional analysis on PGD to show the relevant mechanisms in which PGD participates.

## Methods

### Data resource

Transcriptome RNA-sequencing data of PGD in 374 HCC tissues and 50 non-tumor tissues were downloaded from the TCGA database (https://cancergenome.nih.gov/). Then, clinical information of 370 HCC patients (4 samples without survival information were removed) and PGD DNA methylation expression were also obtained through the TCGA database. In addition, PGD expression and clinical data of 232 HCC samples and 202 non-tumor samples were downloaded from the ICGC database (https://dcc.icgc.org/) to validate the prognostic role of PGD. Furthermore, 242 HCC samples and 239 non-tumor samples from GSE14520 in the Gene Expression Omnibus database (GEO)were also included in the study (18). We summarized the clinicopathological features of HCC patients in these three databases in **Supplementary Table 1**.

### Evaluation of PGD on the prognosis of HCC patients

We evaluated the impact of PGD expression and PGD DNA methylation on overall survival (OS) in HCC patients from the TCGA database, using Kaplan-Meier survival analysis *via* the R package “survival.” A ROC curve was also performed with the R package of “survival ROC”. The prognostic significance of PGD for HCC patients was validated in the ICGC database. Moreover, a meta-analysis with all HCC patients based on the three databases was used to further verify and summarize the prognostic utility of PGD for HCC. We showed the result of a meta-analysis by forest plot, and pooled HR (hazard ratio) was calculated based on the fixed-effect model because there is no obvious heterogeneity (I^2^<50%). Then, univariate Cox proportional hazards regression analysis was conducted based on PGD expression and clinical factors, and factors with p value< 0.05 in univariate Cox regression analysis were included in multivariate proportional hazards regression analysis.

### Immune cell infiltration analysis

The immune cell infiltration of the high PGD expression group and the low PGD expression group was compared based on several algorithms, including ESTIMATE (19), MCP-counter (20), ssGSEA (21) and CIBERSORT methods (22). The immune score and ESTIMATE score of all samples were calculated using the ESTIMATE algorithm. MCP-counter method was applied to evaluate the abundance of CD8+T cells, cytotoxic lymphocytes, myeloid dendritic cells, monocytic lineage cells, neutrophils, NK cells, T cells and endothelial cells. The enrichment scores calculated by ssGSEA method was used to assess 28 types immune cell infiltrations and gene markers of these immune cells were obtained from a previous study (23). Finally, we used CIBERSORT to measure 22 kinds of immune cells in HCC tissues.

In addition, the correlations between PGD expression and the amount of six kinds of immune cell infiltration (CD4 + T cells, CD8 + T cells, B cells, neutrophils, dendritic cells, and macrophages) in HCC were conducted using TIMER (Tumor Immune Estimation Resource) (24). Then, we also explored the relationship between marker genes of immune cells (monocyte, macrophage, Treg cells, dendritic cells, B cells, neutrophils, CD8 + T cells, Th1 cells, NK cells) and PGD to further reflect the relationship between immune cells and PGD. The marker genes of these immune cells are based on the previously published article (25).

### Predicting the response of PGD subgroups to immunotherapy therapy

Currently, tumor immunotherapy has become the focus of cancer treatment. And many factors in the research can predict the response and efficacy of immunotherapy, including tumor mutation burden (TMB) (26), immune checkpoints (27), and human leukocyte antigen (HLA) member expression (28). In order to further evaluate the role of PGD in immunotherapy of HCC, we explored the differences of the expression of immune checkpoints, HLA member expression, TMB and tumor immune dysfunction and exclusion (TIDE) score in the high and low PGD expression subgroups. Among them, TIDE (http://tide.dfci.harvard.edu) is a new computing architecture based on the tumor immune escape mechanism and has been verified to predict efficacy of immunotherapy (29). In addition, 360 cases from TCGA were grouped according to the classification criteria of tumor immune subtypes (30), and we compared the different immune subtypes in the high expression PGD and the low expression PGD group. Finally, according to the grouping of immune subtypes, the response of each immune subtype group to immunotherapy was evaluated according to TIDE scores.

### GSEA gene enrichment analysis

GSEA (Gene Set Enrichment Analysis) is a tool for analyzing genome-wide expression profile chip data (31). The GO (Gene Ontology) term and KEGG (Kyoto Encyclopedia of Genes and Genomes) pathways enriched by PGD are explored in HCC. And KEGG enrichment pathways and GO terms with the false discovery rate p-value (FDR p-value) less than 0.05 were considered significant.

### Statistical analysis

The median PGD expression was regarded as the cutoff value in the three databases. HR values and 95% CI (confidence interval) values used for meta-analysis were obtained and analyzed by SPSS 25. Meta-analysis was performed with STATA 13.0. Additionally, we used R version 3.5.1 to complete the remaining statistical analyses in this study (32). And p-value was two-sided, and the p value < 0.05 was viewed as statistically significant (32).

## Results

### Prognostic value of PGD expression in TCGA database

**Figure 1:**
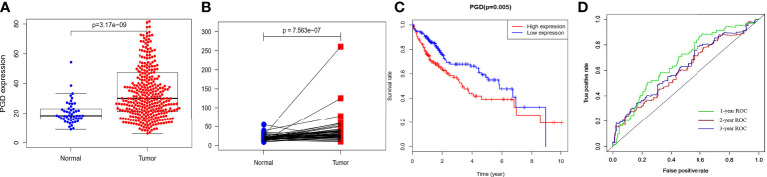
The expression and clinical significance of PGD in TCGA database **(A)** PGD is highly expressed in HCC tissues. **(B)** PGD is highly expressed in HCC tissues by paired analysis. **(C)** Kaplan-Meier curve for the high and low PGD expression group of HCC patients. **(D)** 1-year, 2-year, and 3-year ROC curve of PGD expression.

The RNA-sequencing data of 374 HCC tissues and 50 nontumor tissues were downloaded from TCGA, and we found that PGD expression showed lower expression in nontumor tissues while higher expression in HCC tissues (**Figure 1A**), and then paired analysis further proved the results (**Figure 1B**). Then, HCC patients were grouped into low PGD expression and high PGD expression according to the median expression of PGD. Subsequently, Kaplan-Meier plots showed that low PGD expression (p=0.005, **Figure 1C**) predicted better OS than high PGD expression in HCC patients. The 1-year, 2-year, and 3-year ROC curve analysis of PGD demonstrated that the area under the curve (AUC) was 0.667, 0.624, and 0.603, respectively (**Figure 1D**).

**Figure 2:**
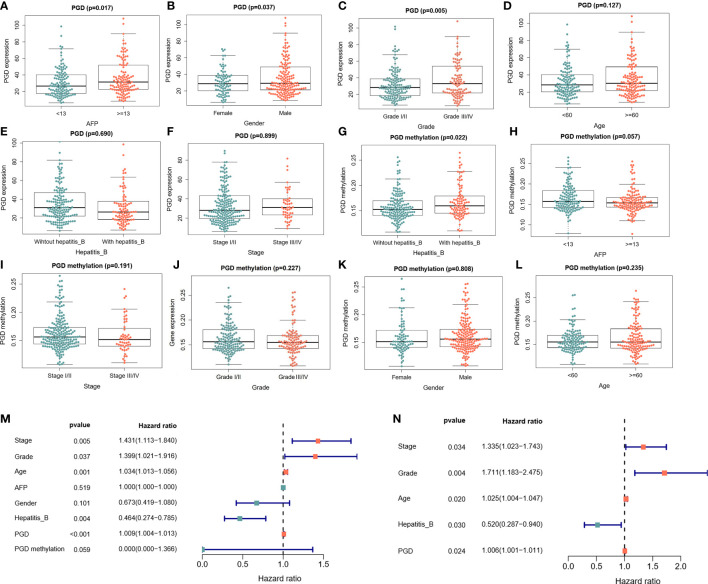
The correlations between PGD expression and PGD methylation with clinical characteristics in TCGA database **(A)** Correlations between PGD expression and AFP. **(B)** Correlation between PGD expression and gender. **(C)** Correlation between PGD expression and grade. **(D)** Correlation between PGD expression and age. **(E)** Correlation between PGD expression and hepatitis **(B) (F)** Correlation between PGD expression and stage. **(G)** Correlation between PGD methylation and AFP. **(H)** Correlation between PGD methylation and stage. **(I)** Correlation between PGD methylation and AFP. **(J)** Correlation between PGD methylation and grade. **(K)** Correlation between PGD methylation and gender. **(L)** Correlation between PGD methylation and age. **(M)** Forest plot for univariate cox analysis. **(N)** Forest plot for multivariate cox analysis. AFP, alpha fetoprotein; Hepatitis B, Hepatitis B virus.

Moreover, the relationships between PGD expression and clinical characteristics were explored. As shown in **Figure 2**, the expression of PGD was closely correlated with AFP (**Figure 2A**, p=0.017), gender (**Figure 2B**, p = 0.037) and grade (**Figure 2C**, p = 0.005). However, the expression of PGD showed no significant relationship with age (**Figure 2D**), hepatitis B virus infection (**Figure 2E**), and stage (**Figure 2F**). And PGD methylation was affected by hepatitis B infection (**Figure 2G**, p=0.022). Then, univariate Cox analysis (**Figure 2M**) and multivariate Cox analysis (**Figure 2N**) with PGD expression and these characteristics were conducted. The results suggested that PGD expression (HR = 1.006, 95% CI = 1.001-1.011, P = 0.024) was independent prognostic factors for OS in HCC patients (**Figure 2N**).

### Verification of the prognostic value of PGD expression in ICGC and GEO database

**Figure 3:**
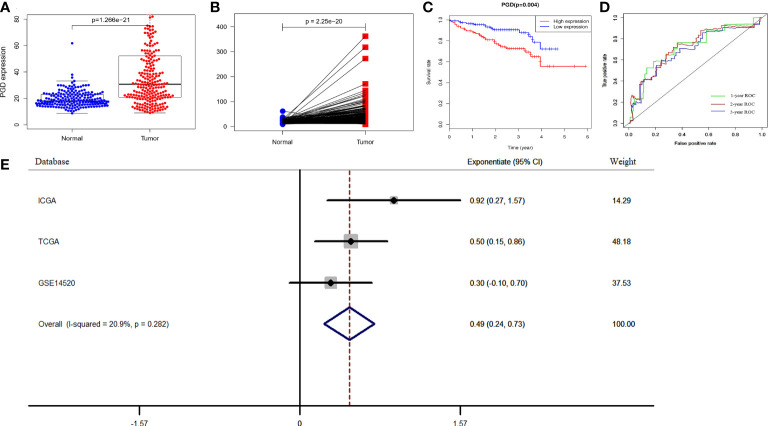
Verification of PGD expression and prognostic power in ICGC and GEO database **(A)** PGD is highly expressed in HCC tissues in ICGC database. **(B)** PGD is highly expressed in HCC tissues by paired analysis in ICGC database. **(C)** Kaplan-Meier curve for the high and low PGD expression group of HCC patients in ICGC database. **(D)** 1-year, 2-year, and 3-year ROC curve of PGD expression in ICGC database. **(E)**Forest plot of low PGD expression with favorable OS in HCC patients based on three datasets (TCGA database, ICGC database and GSE14520 dataset).

To further verify the prognostic efficacy of PGD, we analyzed RNA sequencing data of 232 and 242 patients from the ICGC database and GSE14520 dataset, respectively. And the differential expression of PGD was also confirmed by data from the ICGC database. The results showed that PGD expressed higher in HCC tissue in differential analysis and paired analysis (**Figures 3A, B**). Then, Kaplan-Meier analysis was employed to investigate the association between PGD expression and OS in HCC patients (**Figure 3C**), and we found that low PGD was closely correlated with better OS in the ICGC database (P = 0.004). In addition, the 1-year, 2-year, and 3-year ROC curve analysis showed that the area under the curve (AUC) was 0.724, 0.693, and 0.721, respectively (**Figure 3D**).

According to the results of the meta-analysis based on 848 HCC patients from the above three databases, the pooled HR and 95% CI of the relationship between PGD expression and OS were 0.49(0.24–0.73), and there was no significant heterogeneity between the three databases (I^2^ = 20.9%, P = 0.282, **Figure 3E**). Therefore, we can conclude that low PGD expression is a good predictor of OS in HCC patients.

### The expression of immune cells is different in the high and low PGD expression groups

**Figure 4:**
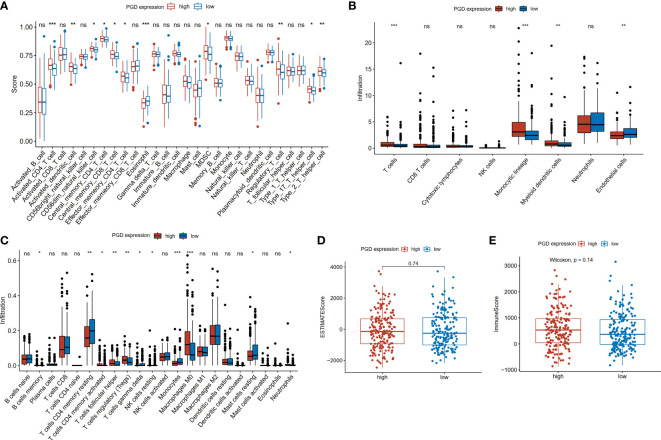
Comparison of immune cells between high and low PGD expression group **(A)** Evaluation of 28 immune cell infiltration using the ssGSEA method. **(B)** Differential infiltration levels of indicated cell types between two PGD expression groups using MCP-counter method. **(C)** Evaluation of 22 immune cell infiltration using the CIBERSORT method. **(D)** ESTIMATE scores in the high and low PGD expression group based on ESTIMATE algorithm. **(E)** Immune scores in the high and low PGD expression group based on ESTIMATE algorithm. *represents p < 0.05, **represents p < 0.01, ***represents p < 0.001. ns represents p>=0.05.

The differences in tumor-infiltrating immune cells between high and low PGD expression groups were compared based on the several algorithms. First, we used the ssGSEA algorithm to estimate the relative quantity of 28 infiltrating immune cells in each tumor sample from the TCGA database. The relative quantity of activated CD4+T cell (p<0.001), activated dendritic cell (p<0.01), CD56 dim natural killer cell (p<0.05), central memory CD8+T cell (CD8+TCM, p<0.05), central memory CD4+T cell (CD4+TCM, p<0.05), effector memory CD4+T cell (CD4+TEM, p<0.05), myeloid-derived suppressor cells (MDSC, p<0.05), regulatory T cell (Tregs, p<0.01), T helper cell 17 (Th17, p<0.05) and T helper cell 2 (Th2, p<0.01) was higher in the high PGD expression group, while the relative quantity of eosinophil (p<0.001)was higher in the low PGD expression group (**Figure 4A**). Second, the abundance of six immune-related cells and two stromal cells was estimated using the MCP-counter algorithm. Compared with the low PGD expression group, the abundance of T cells (p<0.001), monocytic lineage (p<0.001), myeloid dendritic cell (p<0.01) was significantly upregulated in the high PGD expression group, while endothelial cells (p<0.01) were upregulated in the low PGD expression group (**Figure 4B**). Third, we also used the CIBERSORT method to further evaluate the relative fraction of 22 infiltrating immune cells in each tumor sample. The relative fractions of memory B cell (p<0.05), activated memory CD4+T cell (p<0.05), follicular helper T cell (Tfh, p<0.01), Tregs (p<0.01), macrophage M0 (p<0.001) and neutrophils (p<0.05) were significantly elevated in high PGD expression group, while the relative fractions of resting memory CD4+T cell (p<0.01), gamma delta T cells (p<0.05), resting NK cells (p<0.05), monocytes (p<0.001) and resting mast cells (p<0.05) were higher in low PGD expression group (**Figure 4C**). Last, we calculated ESTIMATE score and the immune score based on the ESTIMATE algorithm. The immune score (585.01 vs. 487.17) and ESTIMATE score (-54.58 vs. -72.15) were higher in high PGD expression group compared with low PGD expression group, while the difference showed no statistical difference (**Figures 4D, E**). Furthermore, the differences in tumor-infiltrating immune cells between high and low PGD expression groups were further analyzed in ICGC database and GSE14520 database using the three algorithms (**Supplementary Figures 3D-I**). Moreover, we also analyzed differences in immune cells between patients receiving chemotherapy and those not receiving chemotherapy (**Supplementary Figures 3A–C**). The results indicated that the fractions of activated NK cell and activated dendritic cell were elevated in chemotherapy group using CIBERSORT algorithm.

Furthermore, the correlations between PGD expression and the markers of 9 kinds of immune cells were evaluated in HCC using the TIMER database (**Supplementary Table 2**) and we also showed these correlations adjusted by purity and age in **Supplementary Table 2**. As illustrated in **Supplementary Figure 1**, the results suggested that PGD expression was related with immune markers of neutrophils (**Figure S1A**), macrophage (**Figure S1C**), monocyte (**Figure S1D**), Treg cell (**Figure S1E**), B cells (**Figure S1G**), CD8 + T cells (**Figure S1H**), and dendritic cell (**Figure S1I**) in HCC. However, there are no significant relationships between PGD expression and immune markers of NK cells (**Figure S1B**) and Th1 cells (**Figure S1F**). In addition, we also explored the correlation between PGD mRNA expression and immune cell infiltration based on the TIMER database. As shown in the plots (**Figure S1J**), the expression of PGD was positively correlated with B cells (P <0.0001), CD8+ T cells (P =0.000546), CD4+ T cells (P =0.000131), macrophage (P <0.0001), and neutrophils (P <0.0001) and dendritic cell (P <0.0001) infiltration.

### Patients in the high PGD expression group respond better to HCC immunotherapy

**Figure 5:**
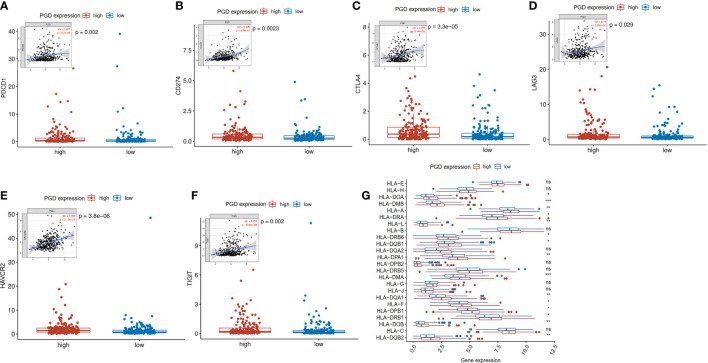
Expression levels of immune checkpoints and HLA members **(A)** Significantly elevated expression level of PDCD1 was found in the high PGD expression group. **(B)** Significantly elevated expression level of CD274 was found in the high PGD expression group. **(C)** Significantly elevated expression level of CTLA4 was found in the high PGD expression group. **(D)** Significantly elevated expression level of LAG3 was found in the high PGD expression group. **(E)** Significantly elevated expression level of HAVCR2 was found in the high PGD expression group. **(F)** Significantly elevated expression level of TIGIT was found in the high PGD expression group. **(G)** The profile of HLA member expression levels indicates an overall enhance in HLAs in the high PGD expression group. *represents p < 0.05, **represents p < 0.01, ***represents p < 0.001, ns represents p>=0.05.

Then, to help explore the application of PGD in immunotherapy, we further explored whether immune checkpoints are differentially expressed between high- and low-expressed PGD groups, including programmed cell death-1(PD1; also known as PDCD1), programmed cell death-Ligand 1(PDL1; also known as CD274), T lymphocyte-associated antigen 4 (CTLA4), lymphocyte activation gene3 (LAG3), T cell membrane protein 3 (TIM3; also known as HAVCR2), and T cell immunoreceptor with Ig and ITIM domains (TIGIT) (33). As exhibited in **Figures 5A–F**, PD1 expression (p=0.002), PDL1 expression(p=0.0023), CTLA4 expression(p<0.0001), LAG3(p=0.029), HAVCR2(p<0.0001), and TIGIT(p=0.002) were significantly higher in the high PGD expression group than in the low PGD expression group. Lack of HLA may impair the ability of cells to present new antigens and lead to immune tolerance (28). Then, we explored expression of HLA members in the high PGD expression and low PGD expression group. The result showed that most HLA members (including HLA-DOA, HLA-DMB, HLA-A, HLA-DRA, HLA-L, HLA-DRB6, HLA-DQB1, HLA-DPA1, HLA-DMA, HLA-DQA1, HLA-F, HLA-DPB1, HLA-DRB1, HLA-DOB, and HLA-DQB2) were higher in high PGD expression group compared with low PGD expression group (**Figure 5G**).

**Figure 6:**
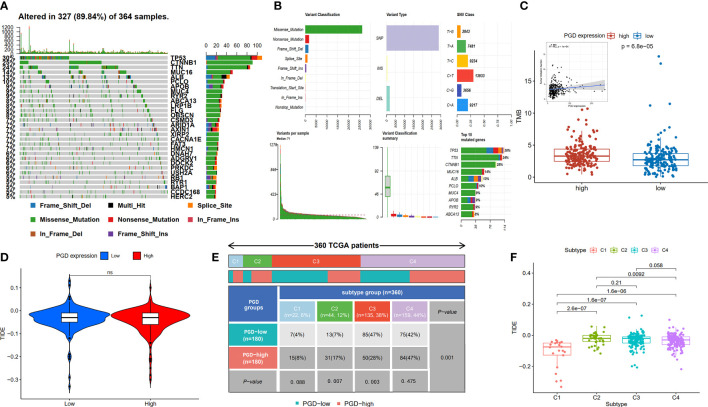
Analysis of TMB expression and TIDE score **(A, B)** Profile of genetic alteration in the TCGA HCC cohort. **(C)**The high PGD expression group exhibits significantly higher TMB than the low PGD expression group. **(D)** The relationship between PGD expression and TIDE. **(E)** The distribution of cases in C1, C2, C3, and C4 immune subtype based on TCGA database. **(F)** The relationship between the four immune subtypes and TIDE. TMB, tumor mutation burden, TIDE, tumor immune dysfunction and exclusion. ns represents p>=0.05.

Furthermore, TMB refers to the number of somatic mutations per one million bases, which is a new feature of cancer and patients with high TMB respond significantly better to immunotherapy. The waterfall diagram revealed the overall status of somatic mutations in TCGA HCC conducted by the MutSigCV algorithm (**Figure 6A**). The findings showed that missense mutations, SNP, and C>T mutation were more common, and the highest mutation frequency was 1270 (**Figure 6B**). As we expected, TMB was significantly higher in high PGD expression group compared to low PGD expression group, which indicated that high PGD expression group patients may respond better to immunotherapy (p<0.001, **Figure 6C**).

The TIDE score could evaluate immune escape and immunotherapy response, and a high TIDE score predicts a poor response for immunotherapy. We showed TIDE score (-0.043 vs. -0.035) was lower in high PGD expression group compared to low PGD expression PGD, while there was no statistical significance (**Figure 6D**). In addition, as shown in **Figure 6E**, detailed information of four immune subtypes, including C1, C2, C3, C4 in the low and high PGD expression group, was exhibited. And we could conclude that there were obvious differences in C2 (p=0.007), C3 (p=0.003) and overall immune subtypes (p=0.001) between the low PGD expression group and the high PGD expression group. Then, the plot suggested that TIDE (**Figure 6F**) was different in the four immune subtypes of HCC, which indicates that different immune subtypes are significantly related to the therapeutic effect of immunotherapy. In short, the above results could provide new ideas and foundations for the immunotherapy of HCC.

### Mechanisms of PGD and PGD DNA methylation in HCC

**Figure 7:**
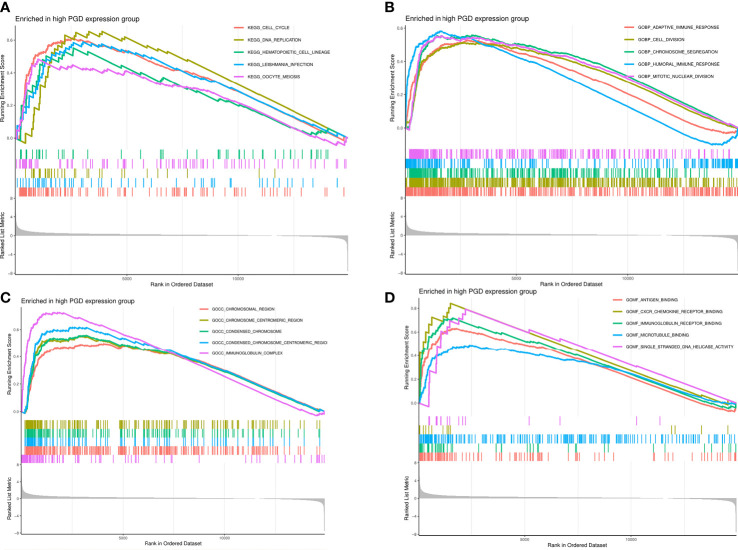
The results of GSEA analysis **(A)** The top 5 significant enrichment KEGG pathways in high PGD expression group. **(B)** The top 5 significant enrichment GO biological processes in high PGD expression group. **(C)** The top 5 significant enrichment GO cellular components in high PGD expression group. **(D)** The top 5 significant enrichment GO molecular functions in high PGD expression group.

In order to gain insight into the potential signaling pathways of PGD in HCC, GSEA analysis was used to determine its possible biological processes and functional role. As shown in **Figure 7A**, we exhibited the top 5 KEGG pathways enriched in PGD high expression group in HCC, including cell cycle, DNA replication, hematopoietic cell lineage, Leishmania infection, and pentose phosphate pathway. In addition, the top 5 gene ontology (GO) biological processes enriched in PGD high expression group were demonstrated in **Figure 7B**, including adaptive immune response, cell division, chromosome segregation, humoral immune response, and mitotic nuclear division. The top 5 GO cellular components enriched in PGD high expression group were chromosomal region, chromosome centromeric region, condensed chromosome, condensed chromosome centromeric region, and immunoglobulin complex (**Figure 7C**). And the top 5 GO molecular functions were antigen binding, CXCR chemokine receptor binding, DNA replication origin binding, immunoglobulin receptor binding, and microtubule binding (**Figure 7D**). Additionally, Hong et al. have shown that phosphatase and tensin homolog (PTEN) inhibits PPP pathway in human liver tumors. PTEN inhibits glucose consumption and biosynthesis through PPP (34). Therefore, we also explored the expression of PTEN in the database, but we did not find that PTEN was down-regulated in HCC tissues (**Supplementary Figure 4**).

**Figure 8:**
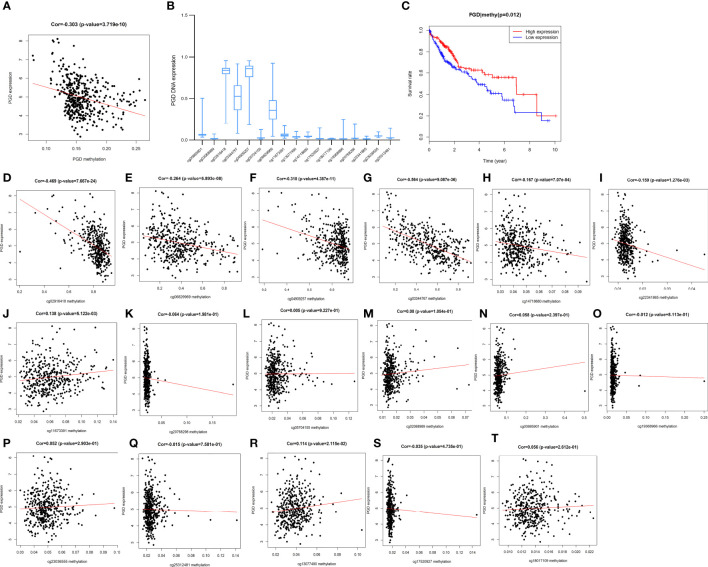
Correlations between PGD expression and PGD methylation in TCGA database **(A)** The expression of PGD was negatively regulated by PGD DNA methylation. **(B)** The distribution of 17 PGD DNA promoter CpG sites. **(C)** Kaplan-Meier curve for the PGD hypermethylation and hypomethylation group of HCC patients. **(D-T)** Correlations between PGD expression and PGD DNA promoter CpG sites in HCC patients, including cg02916418 site **(D)**, cg06829969 site **(E)**, cg04909257 site **(F)**, cg03344767 site **(G)**, cg14718680 site **(H)**, cg22341865 site **(I)**, cg11673391 site **(J)**, cg20768298 site **(K)**, cg05704155 site **(L)**, cg0208989 site **(M)**, cg00885901 site **(N)**, cg19368966 site **(O)**, cg23036555 site **(P)**, cg25312481 site **(Q)**, cg13077490 site **(R)**, cg17520927 site **(S)**, and cg18017109 site **(T)**.

Furthermore, PGD expression was shown to be negatively correlated with PGD DNA methylation (P <0.0001) (**Figure 8A**). Moreover, 17 PGD CpG sites were exhibited in **Figure 8B**. Next, the correlations between PGD expression and PGD gene methylation at these 17 CpG sites (**Figures 8D–T**) were explored by Pearson correlation analysis. We could see that 6 CpG sites, including cg02916418 (**Figure 8D**), cg06829969 (**Figure 8E**), cg04909257 (**Figure 8F**), cg03344767 (**Figure 8G**), cg14718680 (**Figure 8H**), and cg22341865 (**Figure 8I**), were correlated with the expression of PGD, while PGD gene methylation at the remaining CpG sites showed no relationship.

Furthermore, HCC patients with high PGD methylation levels showed longer OS (p=0.012, **Figure 8C**) according to Kaplan-Meier curve analysis. In addition, we investigated the predictive values of the 17 PGD CpG sites in HCC patients. As shown in **Supplementary Figure 2**, hypomethylation of cg06829969 CpG sites (**Figure S2A**, p=0.002) and hypermethylation of cg13077490 CpG sites (**Figure S2B**, p=0.027) were correlated with poorer OS among patients with HCC, and the remaining CPG sites showed no significant relationship with the prognosis of HCC patients.

## Discussion

In our research, low PGD expression in the TCGA database suggested a better prognosis for HCC patients, and this conclusion was also verified in the ICGC database. We found that the level of immune cell infiltration was significantly different between low and high PGD expression group, in which most immune cell infiltrations were higher in high PGD expression group. At the same time, the results revealed that high PGD expression group responded better to immunotherapy. In summary, this study provided a new research perspective and research foundation for PGD in the prognosis prediction of HCC and the evaluation of immunotherapy effect in HCC.

As far as we know, there is no research on the prognostic value of PGD expression and PGD immune infiltrating in HCC. We found that the expression of PGD in HCC tissues is higher than that in adjacent non-tumor tissues, which is consistent with the results of a previous study (12). PGD upregulation has been proved to be related to the occurrence and development of a variety of tumors, including breast cancer, colon cancer, cervical cancer, and so on, and this may be due to the coordinated regulation of PGD involved in anabolic and redox (35–39). In these studies, Hu Chen, etc., confirmed a correlation between the high expression of PGD and HCC (12), but there is currently a lack of research on the prognosis of PGD for HCC and related research on its function in HCC. In this study, we used three public databases to explore the relationship between PGD expression and the survival of HCC patients. According to the results of survival analysis and multivariate Cox regression analysis, we proved that PGD expression could be used as an independent prognostic factor for predicting the prognosis of HCC. In addition, a meta-analysis of 848 patients based on the three databases also further revealed that low PGD expression predicts a better prognosis for HCC patients. Moreover, we also found PGD expression was correlated with AFP levels. PGD is a key enzyme in the PPP, which is important in cancer, and activation of this pathway induces tumorigenesis (6). Tumor cells increase nucleotide synthesis through the PPP process, thereby increasing the growth rate of tumor cells (6). Furthermore, AFP is thought to be significantly associated with the growth and development of HCC (40). Our findings suggest that patients with high AFP expression exhibited high levels of PGD expression, which may be a manifestation of PGD promoting HCC growth and development through the PPP pathway.

Increasing evidence suggests that immune cell infiltration plays an important role in the development and progression of HCC (41). In the present study, we discovered that immune score and ESTIMATE score were higher in high PGD expression group compared to low PGD expression group. Then, based on ssGSEA method, high PGD expression was correlated with several types of T cells, including activated CD4+T cell, CD8+TCM, CD4+TCM, CD4+TEM, Tregs, Th17 and Th2. And the CIBERSORT algorithm further confirmed that there was a significant difference in the abundance of immune cells between the high and the low PGD expression group. Similarly, previous study showed that blocking PGD in oxidized PPP will reduce the inhibitory function of Tregs and the ability of Tregs to differentiate into Th1, Th2 and Th17 phenotypes (17). In addition, Saeed Daneshmandi et al. uncovered that PGD might play an important role in regulating CD8+ T cell activation and differentiation. Blocking PGD would lead to faster and better development of effector functions in the stimulated naive CD8+ T cells, thereby enhancing the ability to inhibit tumor growth *in vivo (* 16). These results indicated that different immune cells infiltration levels may partly explain the difference in survival between the patient groups with high and low PGD expression. Additionally, most immunotherapy works through immune cells, in which immune checkpoint inhibitors enhance T cell response to tumors by blocking immune checkpoint receptors (42). However, we lack information about immunotherapy and cannot analyze changes in immune cells in patients who receive immunotherapy, which needs to be further analyzed and validated in clinical patients.

Given that we have demonstrated that PGD is significantly related to survival of HCC patients and immune cells level, it may contribute to select patients who respond better to immunotherapy and improve patient treatment options. We evaluated the association between PGD expression and several immunotherapy biomarkers, including immune checkpoint genes, TMB (43), HLA (28) and TIDE. High levels of immune checkpoint genes and TMB are generally considered predictive biomarkers for patients who are suitable for immunotherapy. We found that compared with the low PGD expression group, the expression level of PD1, PDL1, CTLA4, LAG3, HAVCR2, and TIGIT in the high PGD expression group was significantly upregulated. Consistently, high PGD expression group tend to show higher TMB levels than low PGD expression group. These results indicate that the high PGD expression group is more likely to respond better to immunotherapy. In addition, we also found that most HLA genes were higher in the high PGD expression group. Decreased HLA expression may weaken the ability of cells to present new antigens and lead to immune escape (28). Additionally, PGD activation increased the chemo- and immune-resistance of renal cell carcinoma (RCC) cells, and PGD inhibition made RCC cells sensitive to chemotherapy and immunotherapy (such as IFN-α) (44). Similarly, we found that high PGD expression indicated a better response to immunotherapy. Overall, these results indicated that PGD affected immune cell infiltrating and correlated with the therapeutic effect of immunotherapy in HCC. Thus, it can be used as a potential target for HCC immunotherapy.

According to recent studies, epigenetic disorders are an important driving factor for human malignancies, and of course, HCC is no exception. Among these studies, the role of DNA methylation in the occurrence and development of HCC is one of the hot spots (45, 46). However, there is currently a lack of exploration of the relationship between PGD methylation and the prognosis of HCC. We have confirmed for the first time that PGD hypermethylation is significantly associated with a favorable prognosis of HCC patients. In addition, it was also discovered for the first time that PGD expression and PGD methylation status were negatively correlated in HCC. Then, we further explored the correlation between PGD methylation at specific CpG sites and PGD expression. Among them, the methylation level of 6 CpG sites (including cg02916418, cg06829969, cg04909257, cg03344767, cg14718680, and cg22341865) was significantly correlated with PGD. Moreover, PGD hypermethylation at cg06829969 CpG sites and hypomethylation at cg13077490 CpG sites were correlated well with better prognosis in HCC patients. Taken together, PGD expression was negatively related to PGD methylation, and PGD methylation status might be a promising predictive factor for OS in HCC patients.

## Conclusion

In summary, PGD shows great potential as a prognostic biomarker and predictor of immunotherapy for patients with HCC. This study is the first to demonstrate that high PGD expression is correlated with the favorable therapeutic effect of immunotherapy, and low PGD expression indicates a better prognosis in HCC patients. In addition, we found that PGD expression is elevated in HCC tissues and is negatively regulated by the level of PGD DNA methylation. And PGD may play an important role in immunotherapy and can be an effective prognostic biomarker for HCC patients, but the related regulatory pathways require further experimental exploration.

## Data availability statement

The data that support the findings of this study are available in The Cancer Genome Atlas database at https://cancergenome.nih.gov, TIMER database at https://cistrome.shinyapps.io/timer, Gene Expression Omnibus database at https://www.ncbi.nlm.nih.gov/geo under the accession number GSE14520, and International Cancer Genome Consortium database at https://dcc.icgc.org.

## Ethics statement

The studies involving human participants were reviewed and approved by All procedures performed in studies involving human participants were in accordance with the ethical standards of the institutional and/or national research committee and with the 1964 Helsinki declaration and its later amendments or comparable ethical standards. For this type of study formal consent is not required. Written informed consent for participation was not required for this study in accordance with the national legislation and the institutional requirements.

## Author contributions

TL, JQ and QZ contributed to the design of the study protocol. Data collection was performed by HW and LW. Data analysis was performed by LZ, JZ, and CQ. The first draft of the manuscript was written by TL and all authors commented on previous versions of the manuscript. All authors contributed to the article and approved the submitted version.

## Funding

This study was supported by National Natural Science Foundation of China (No.82160124; 82100641; 82103075).

## Acknowledgments

The authors thank all data providers, patients, investigators, The Cancer Genome Atlas database, TIMER database, International Cancer Genome Consortium database, Gene Expression Omnibus database and institutions involved in these studies.

## Conflict of interest

The authors declare that the research was conducted in the absence of any commercial or financial relationships that could be construed as a potential conflict of interest.

## Publisher’s note

All claims expressed in this article are solely those of the authors and do not necessarily represent those of their affiliated organizations, or those of the publisher, the editors and the reviewers. Any product that may be evaluated in this article, or claim that may be made by its manufacturer, is not guaranteed or endorsed by the publisher.
