# Supplementary material for: Phosphogluconate dehydrogenase is a predictive biomarker for immunotherapy in hepatocellular carcinoma
Source: Front Oncol. 2022 Oct 20;12:993503. doi: 10.3389/fonc.2022.993503 (PMC9632284; doi:10.3389/fonc.2022.993503)
Supplement: Supplementary file 1 [file DataSheet_1.pdf]

Supplementary table 1. clinicopathological features of HCC patients in the three data sets

Characteristics	TCGA database	ICGC database	GSE14520
Median age, IQR, year	61(51,69)	68.5(62,74)	50(43,58)
Gender			
Male, n	249	171	211
Female, n	121	61	31
Median OS, IQR, year	1.52 (0.88,2.92)	2.14(1.40,3.00)	4.30(1.35,4.80)
grade, n			
G1/G2	232	NA	NA
G3/G4	133	NA	NA
Stage, n			
I/II	256	142	174
III/IV	90	90	51
AFP			
<13	126	NA	NA
⩾13	129	NA	NA
Hepatitis B infection			
With	97	NA	NA
Without	158	NA	NA
Chemotherapy			
yes	33	NA	NA
PGD expression, IQR	30.13(21.30,47.62)	30.64 (20.78,51.60)	6.69(5.96,7.53)

NA represents data is unavailable; OS represents overall survival.

Supplementary table 2. Correlations between gene markers of immune cells and PGD

Cell type	Gene markers	HCC					
		None		Purity		Age	
		COR	P	COR	P	COR	P
B cells	FCRL2	0.140	0.007	0.094	0.083	0.142	0.006
	CD19	0.209	<0.001	0.160	0.003	0.211	<0.001
	MS4A1	0.046	0.380	-0.033	0.547	0.039	0.457
CD8+ T cells	CD8A	0.166	0.001	0.097	0.071	0.163	0.002
	CD8B	0.160	0.002	0.106	0.049	0.158	0.002
Neutrophils	FCGR3B	0.266	<0.001	0.250	<0.001	0.269	<0.001
	CEACAM3	0.161	0.002	0.144	0.007	0.159	0.002
	SIGLEC5	0.297	<0.001	0.267	<0.001	0.295	<0.001
	FPR1	0.321	<0.001	0.314	<0.001	0.317	<0.001
	CSF3R	0.298	<0.001	0.281	<0.001	0.301	<0.001
	S100A12	0.044	0.400	-0.002	0.974	0.041	0.436
Macrophages	CD68	0.346	<0.001	0.335	<0.001	0.341	<0.001
	CD84	0.373	<0.001	0.358	<0.001	0.370	<0.001
	CD163	0.303	<0.001	0.269	<0.001	0.296	<0.001
	MS4A4A	0.328	<0.001	0.313	<0.001	0.324	<0.001
Dendritic cells	CD209	0.353	<0.001	0.336	<0.001	0.347	<0.001
NK cells	KIR3DL3	0.002	0.970	-0.042	0.438	0.001	0.991
	NCR1	0.104	0.046	0.089	0.099	0.099	0.058
Th1 cells	TBX21	0.101	0.051	0.037	0.490	0.095	0.069
Treg	FOXP3	0.182	<0.001	0.183	0.001	0.167	0.001
	CCR8	0.363	<0.001	0.336	<0.001	0.358	<0.001
Monocyte	C3AR1	0.366	<0.001	0.353	<0.001	0.362	<0.001
	CD86	0.355	<0.001	0.343	<0.001	0.353	<0.001
	CSF1R	0.321	<0.001	0.307	<0.001	0.315	<0.001

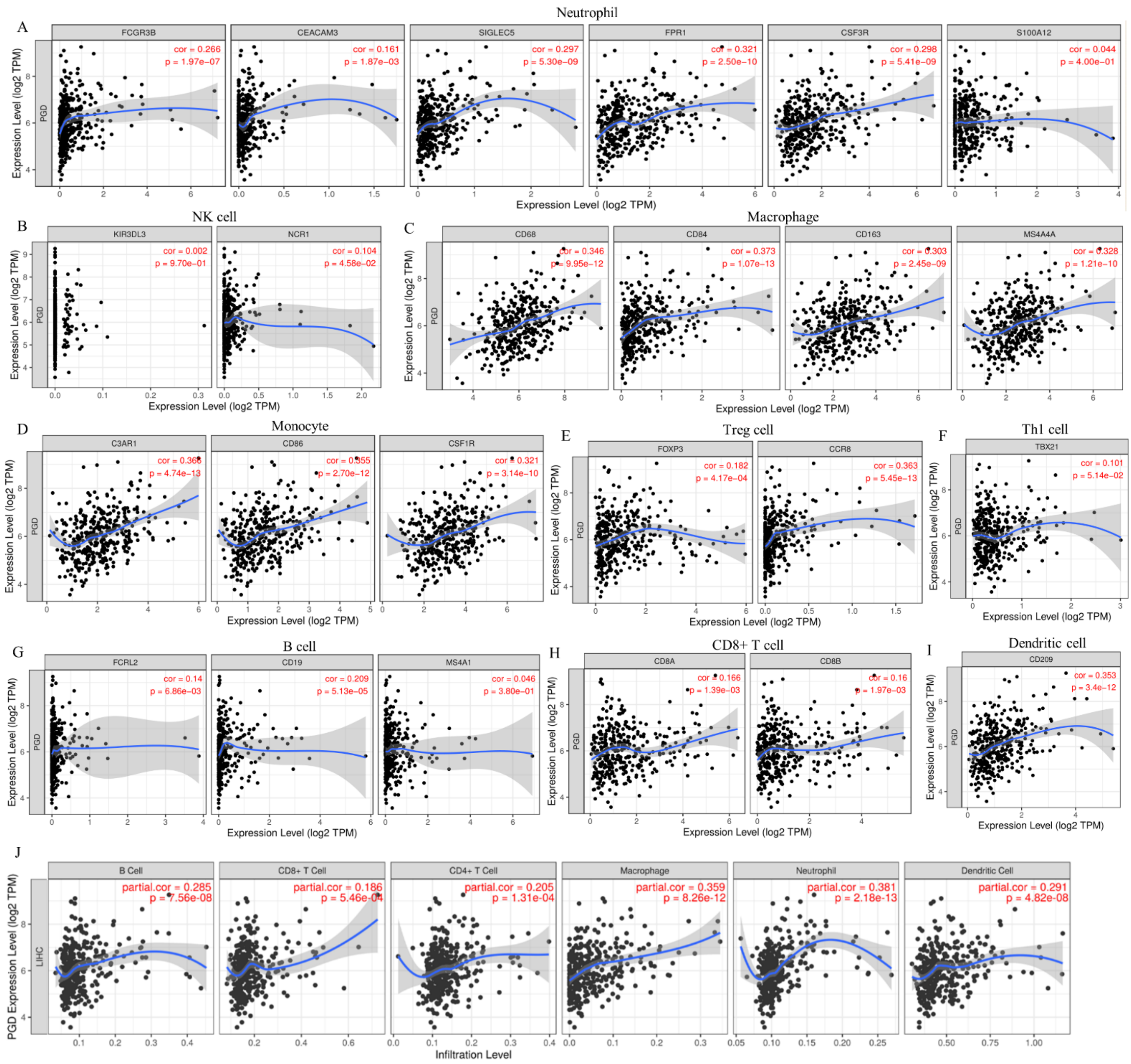

Supplementary figure 1. Correlation between PGD expression and gene markers of immune cells, including neutrophil (A), NK cell (B), macrophage (C), monocyte (D), Treg cell (E), Th1 cell (F), B cell (G), CD8+ T cell (H), and dendritic cell (I). (J)Relationships between PGD expression and 6 types immune cells infiltration after adjusted by purity, including B cell, CD+8 T cell, CD+4 T cell, macrophage, neutrophil and dendritic cell.

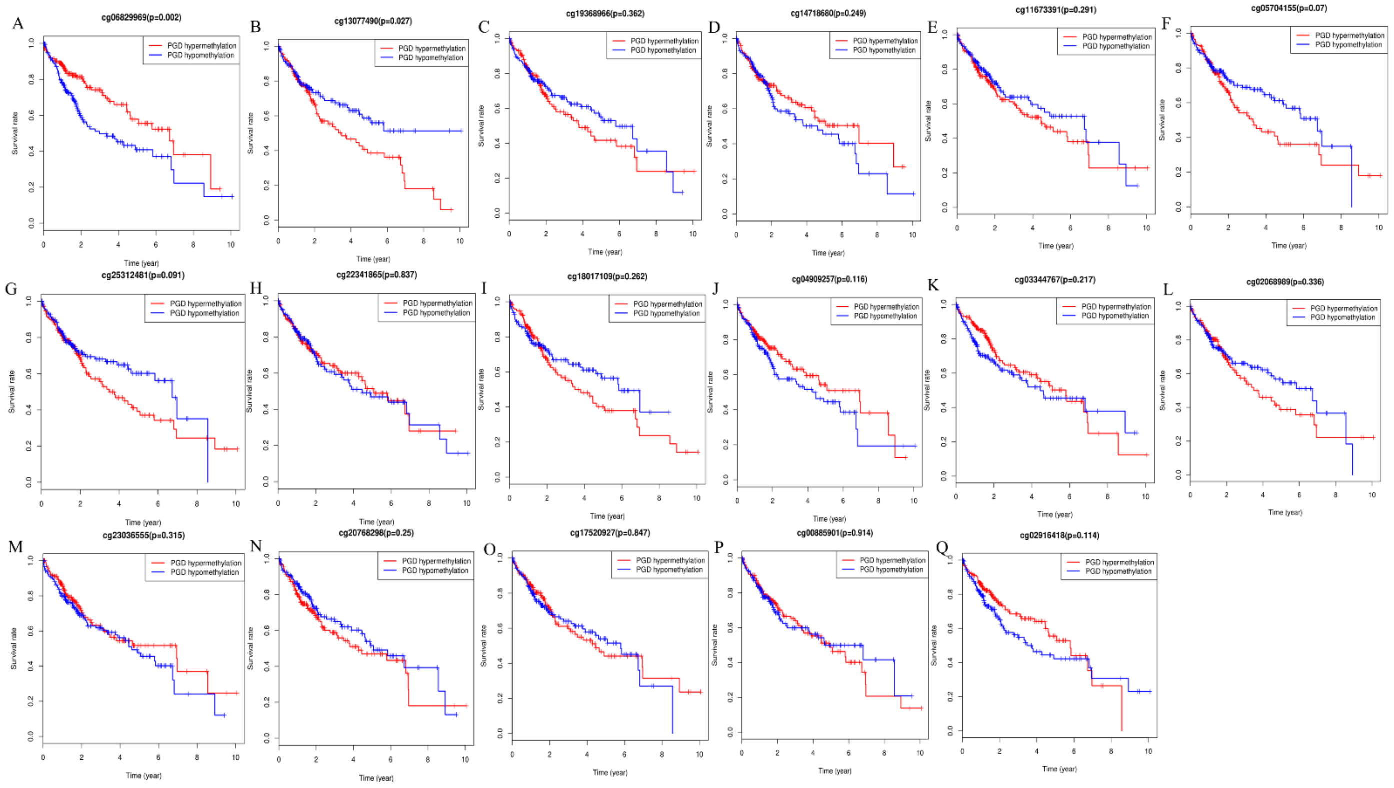

Supplementary figure 2. Kaplan-Meier curves of low and high PGD DNA promoter CpG sites in HCC patients. Kaplan-Meier curve of cg06829969 site (A), cg13077490 site (B), cg19368966 site (C), cg14718680 site (D), cg11673391 site (E), cg05704155 site (F), cg25312481 site (G), cg22341865 site (H), cg18017109 site (I), cg04909257 site (J), cg03344767 site (K), cg02068989 site (L), cg23036555 site (M), cg20768298 site (N), cg17520927 site (O), cg00885901 site (P), and cg02916418 site (Q) in TCGA database.

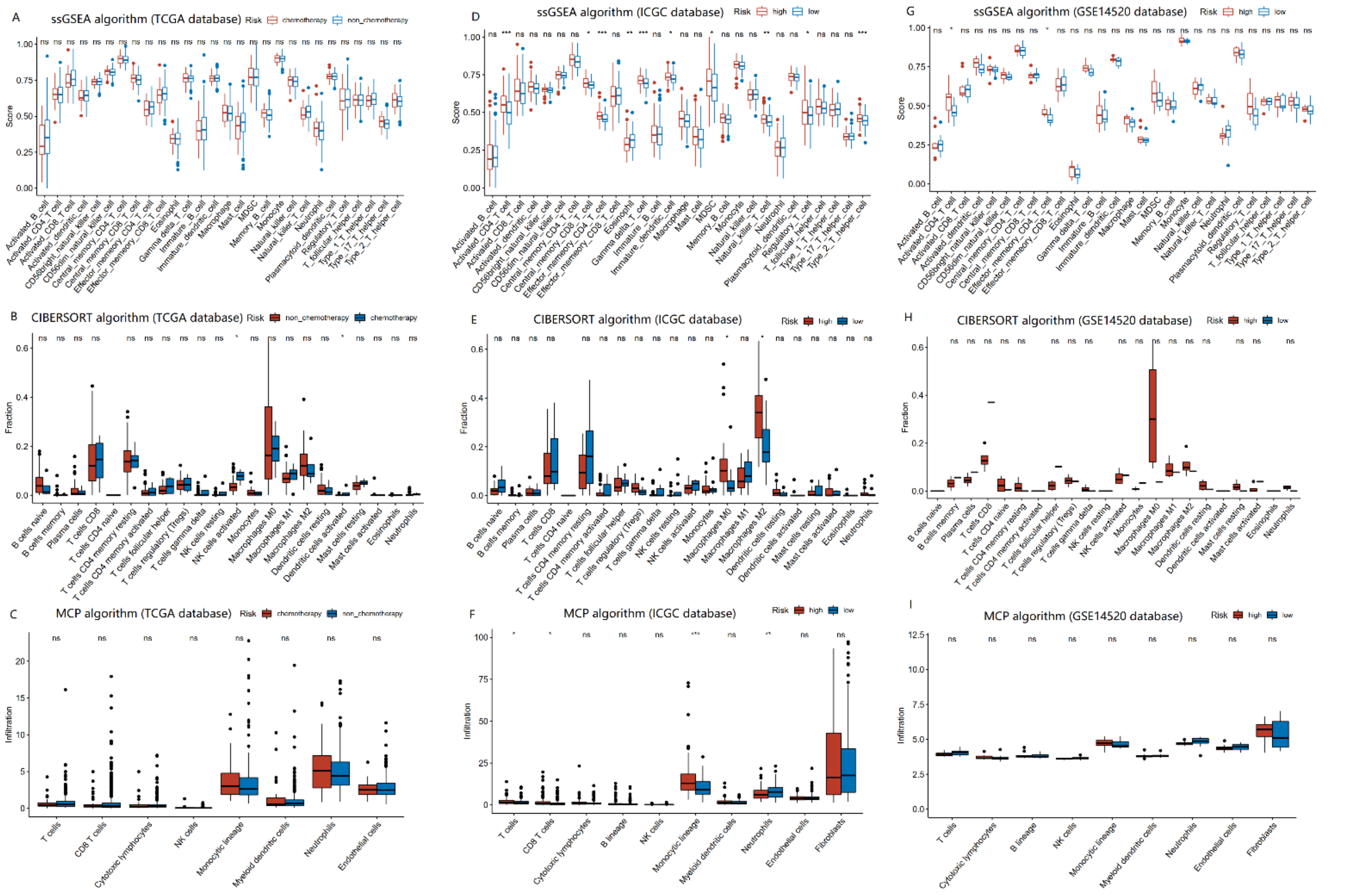

Supplementary figure 3. (A-C) Comparison of immune cells between chemotherapy and non-chemotherapy HCC patients in TCGA database. (A)Evaluation of 28 immune cell infiltration using the ssGSEA method. (B) Differential infiltration levels of indicated cell types between two PGD expression groups using CIBERSORT method. (C) Evaluation of 22 immune cell infiltration using the MCP-counter method.

(D-I) Comparison of immune cells between high and low PGD expression group in ICGC and GEO databases. (D)Evaluation of 28 immune cell infiltration using the ssGSEA method in ICGC database. (E) Differential infiltration levels of indicated cell types between two PGD expression groups using CIBERSORT method in ICGC database. (F) Evaluation of 22 immune cell infiltration using the MCP-counter method in ICGC database. (G)Evaluation of 28 immune cell infiltration using the ssGSEA method in GSE14520 database. (H) Differential infiltration levels of indicated cell types between two PGD expression groups using CIBERSORT method GSE14520 database. (I) Evaluation of 22 immune cell infiltration using the MCP-counter method GSE14520 database.

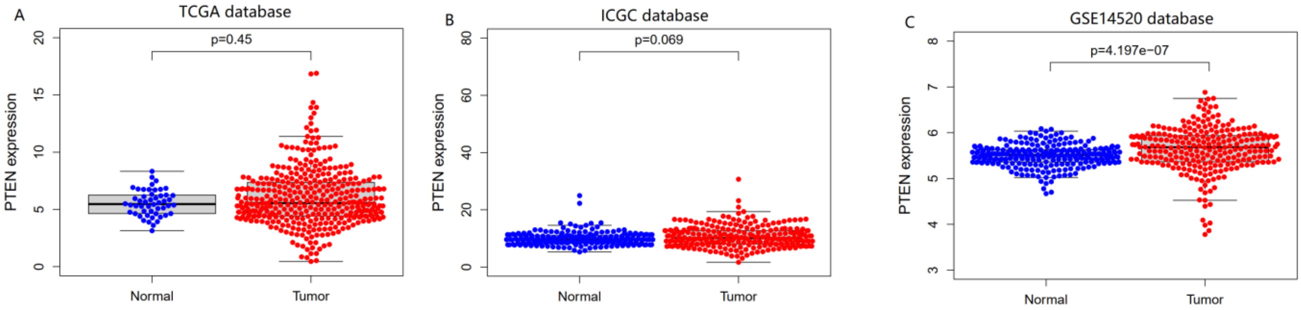

Supplementary figure 4. The expression of PTEN in HCC patients in TCGA, ICGC and GSE14520 data sets.
